# Novel mutation points to a hot spot in *CDKN1C* causing Silver–Russell syndrome

**DOI:** 10.1186/s13148-020-00945-y

**Published:** 2020-10-19

**Authors:** Gerhard Binder, Julian Ziegler, Roland Schweizer, Wisam Habhab, Tobias B. Haack, Tilman Heinrich, Thomas Eggermann

**Affiliations:** 1 Pediatric Endocrinology, University Children’s Hospital, Hoppe-Seyler-Strasse 1, 72076 Tübingen, Germany; 2 Institute of Medical Genetics and Applied Genomics, University of Tübingen, Tübingen, Germany; 3 Institute of Human Genetics, Medical Faculty, RWTH Aachen University, Aachen, Germany

**Keywords:** CDKN1C, Growth retardation, Silver–Russell syndrome

## Abstract

**Background:**

Pathogenic *CDKN1C* gain-of-function variants on the maternal allele were initially reported as a cause of IMAGe syndrome characterized by intrauterine growth retardation, metaphyseal dysplasia, primary adrenal insufficiency and genital anomalies. Recently, a maternally inherited *CDKN1C* missense mutation (p.Arg279Leu) was identified in several members of a single family clinically diagnosed with Silver–Russell syndrome (SRS) but without adrenal insufficiency. Thereafter, two half siblings from UK with familial SRS were described who carried the same mutation. This specific amino acid change is located within a narrow functional region containing the mutations previously associated with IMAGe syndrome.

**Results:**

Here, we describe a third familial case with maternally inherited SRS due to a missense variant affecting the same amino acid position 279 but leading to a different amino acid substitution (p. (Arg279Ser)). The two affected family members (mother and son) presented with the complete SRS phenotype (both Netchine–Harbison CSS score 5 of 6) but without body asymmetry or adrenal insufficiency.

**Conclusions:**

In comparison with loss-of-function genomic *IGF2* mutations, *CDKN1C* gain-of-function mutations are a less frequent cause of SRS and seem to affect a cluster of few amino acids.

## Background

The cyclin-dependent kinase inhibitor 1C (CDKN1C) is a down-regulator of cell proliferation; CDKN1C inhibits the cyclin/CDK complexes of the G1 phase (for review: [1]). It is encoded by the imprinted *CDKN1C* gene on 11p15.5 which is expressed from the maternal allele only [1]. The gene product binds to the cyclin/CDK complex by its C-terminal PCNA-binding domain and exhibits activity by the N-terminal CDK inhibitor domain which is linked to the binding domain by the central PAPA domain [1]. Loss-of-function variants of the maternal allele have been associated with phenotypes of the Beckwith–Wiedemann spectrum (BWSp) [2]. Gain-of-function variants have been identified in individuals with IMAGe syndrome [3], in two families with Silver–Russell syndrome (SRS) [4, 5] and in a third family with an undefined short stature syndrome, and early adult onset diabetes mellitus [6].

BWSp is characterized by a congenital overgrowth phenotype with additional features including macroglossia, exomphalos, lateralized overgrowth and hyperinsulinism. It is an imprinting disorder caused by diverse genetic and epigenetic defects within the two imprinting centers (IC1, IC2) in 11p15.5 encompassing the coding genes *IGF2*, *H19*, *CDKN1C* and *KCNQ1*. In 5% of sporadic and 20% of familial BWS cases, genomic *CDKN1C* variants are detected that are distributed over the whole coding region and are predicted to cause loss of function [2].

IMAGe syndrome is a very rare disorder with intrauterine growth restriction, metaphyseal dysplasia, adrenal hypoplasia and insufficiency as well as genital anomalies. In children with IMAGe syndrome, variants of the maternal *CDKN1C* allele narrowly cluster within five amino acids of the PCNA-binding domain and are thought to confer gain of function [3, 7].

SRS is an imprinting disorder with congenital growth retardation, relative macrocephaly, body asymmetry, prominent forehead, low BMI and severe postnatal growth failure resembling part of the features of IMAGe syndrome [8]. The two major epigenetic causes of SRS are hypomethylation of the IC1 on 11p15.5 (50% of cases) [9] and maternal uniparental disomy of chromosome 7 (upd(7)mat) (10%) [10]. In some individuals with SRS, different alterations of the 14q32.2 imprinted region were detected, changes that were previously associated with Temple syndrome [11]. Rarely, pathogenic variants of *IGF2*, *HMGA2* and *PLAG1* are observed [12, 13]. In addition, two familial SRS cases were described with the identical missense mutation of *CDKN1C* within the PCNA-binding domain (NM_000076.2: c.836G>T; p.(Arg279Leu)) [4, 5]. In contrast to IMAGe syndrome, adrenal insufficiency and metaphyseal dysplasia were absent, but the variant was located within the cluster of IMAGe mutations [4, 5]. Very recently, a sporadic case with SRS having the novel *CDKN1C* variant p.(Arg316Gln) was described by Inoue et al., who performed multigene sequencing in 92 Japanese patients with unexplained SRS [14].

Here, we describe in detail a familial SRS case caused by a novel *CDKN1C* missense variant affecting the same amino acid as in the two SRS families previously reported but leading to a different amino acid substitution (Table 1).

**Table 1:** Characteristics of family members with/out *CDKN1C* mutation

Individual; sex	IV,1; m	IV,2; f	III,2; f	III,3; m	II,2; f	II,3; f	I,1; m
Diagnosis of SRS	Y	N	Y	N	N	N	N
NHCSS score	5/6	0/6	5/6	0/6	0/6	0/6	na
Genotype	C/A	C/C	C/A	C/C	C/A	C/C	C/C
Weeks of gestation; w	33.1	37.9	40.0	39.0	40.0	40.0	na
Birth length; cm; SDS	31.5; − 5.74	49; − 0.01	41; − 5.38	54; + 1.92	52; + 1.04	52; + 1.04	na
Birth weight; g; SDS	770; − 4.26	2485; − 1.75	1560; − 5.21	4220; + 1.59	2800; − 1.73	3900; + 0.87	na
Head CCF at birth; cm; SDS	25.5; − 3.54	30.0; − 2.87	31.0; − 2.14	36.0; + 0.83	na	35.0; + 0.35	na
Head CCF at approx. 2 years; cm; SDS	46,0; − 2.20	na	44.3; − 2.40	na	na	na	na
Adult height; cm	na	na	140.0	185.0	158.8	170	165
Relative macrocephaly	Y	na	Y	na	na	na	na
Prominent forehead	Y	N	Y	N	N	N	na
Feeding difficulties	Y	N	Y	N	N	N	na
Body asymmetry	N	N	N	N	N	N	na
ACTH; pmol/l	3.5	nd	nd	nd	5.3	nd	nd
Cortisol^a^; nmol/l	265	nd	309	nd	204	nd	nd

*NHCSS* Netchine–Harbison clinical scoring system [8], *Y* yes, *N* no, *na* not available, *SDS* standard deviation score, *nd* not done

^a^Serum cortisol and ACTH were measured before 10 a.m.

## Results

The index patient and his mother underwent routine molecular genetic diagnostics, thereby IC1 hypomethylation, upd(7)mat and 14q32.2 alterations were excluded. Exome sequencing revealed a novel heterozygote *CDKN1C* (NM_000076.2) c.835C>A variant predicting the substitution of the evolutionary highly conserved arginine by serine (p.(Arg279Ser)) [3]. This variant is absent from public databases (dbSNP, gnomAD) as well as from > 5000 in-house exome datasets from individuals with unrelated phenotype. In silico analysis predicts the variant as pathogenic (e.g., SIFT (v6.2.1): Deleterious (score: 0, median sequence conservation: 3.26), MutationTaster: disease causing (prob: 0.943)) [15, 16]. According to the American College of Medical Genetics (ACMG) guidelines, the formal criteria PS1, PS3, PS4 and PP1 are fulfilled; therefore, the variant can be characterized as pathogenic [17].

**Fig. 1:**
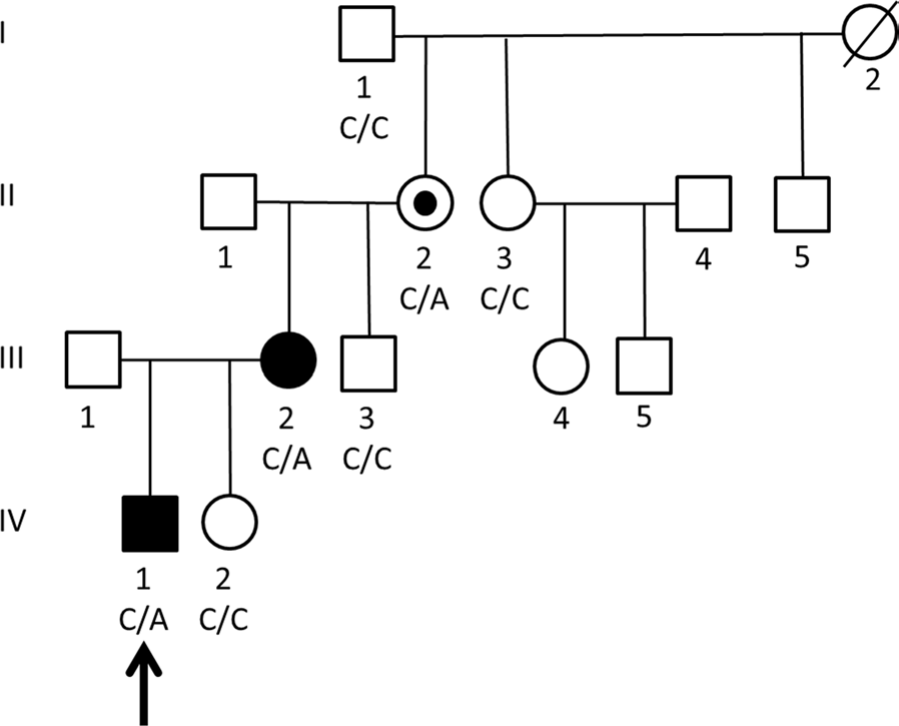
Pedigree of the family. The two affected family members (index IV,1 and mother III,2) carry the mutation c.835C > A as well as the unaffected maternal grandmother (II,2) who is likely to have the mutation de novo. The arrow indicates the index patient

The male index patient and his mother are affected (Fig. 1; pedigree). The maternal grandmother of the index patient is an asymptomatic carrier of the variant. The great-grandfather is not a carrier of the variant. The great-grandmother had deceased, and no material was available for genetic analysis. Testing of the healthy younger sister of the index patient (now one year of age) showed wild-type sequences. The height of the healthy father (III,1) of the index patient is 176.2 cm.

The index patient (IV,1) was born at 33.1 weeks of gestation by caesarian section because of a pathological cardiotocogram. Intrauterine growth restriction and oligohydramnios were evident during the last weeks of gestation. He was severely small for gestational age: Birth weight was 770 g (− 4.3 SDS) and birth length 31.5 cm (− 5.7 SDS). He had relative macrocephaly at birth with a head circumference of 25.5 cm (− 3.5 SDS). Infancy was complicated by feeding difficulties. He reached the motor milestones delayed with free walking at the age of 21 months. Speech development was normal. A bilateral cryptorchidism required orchidopexy at the age of 15 months.

**Fig. 2:**
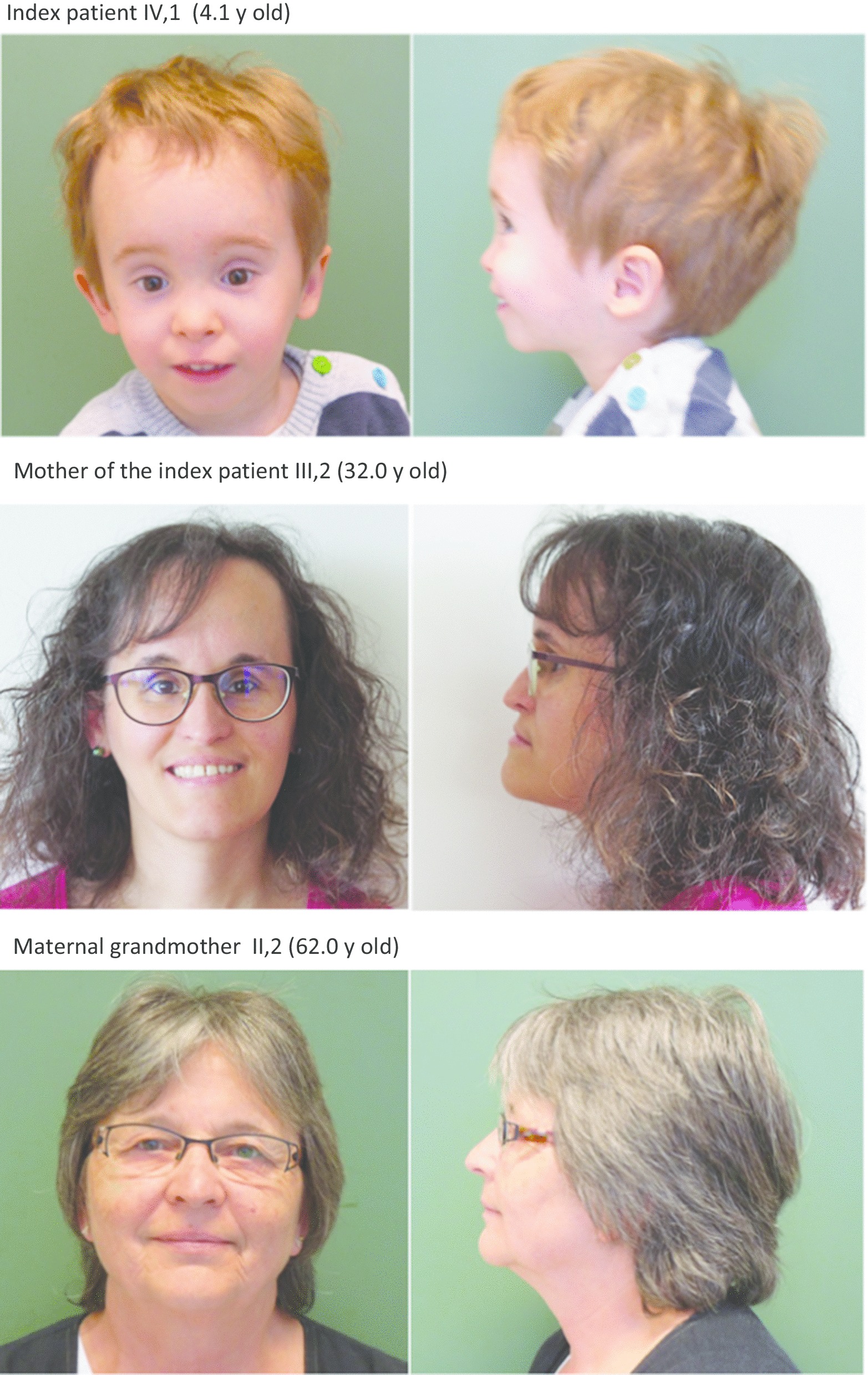
Photographs of index patient, his affected mother and his unaffected maternal grandmother

**Fig. 3:**
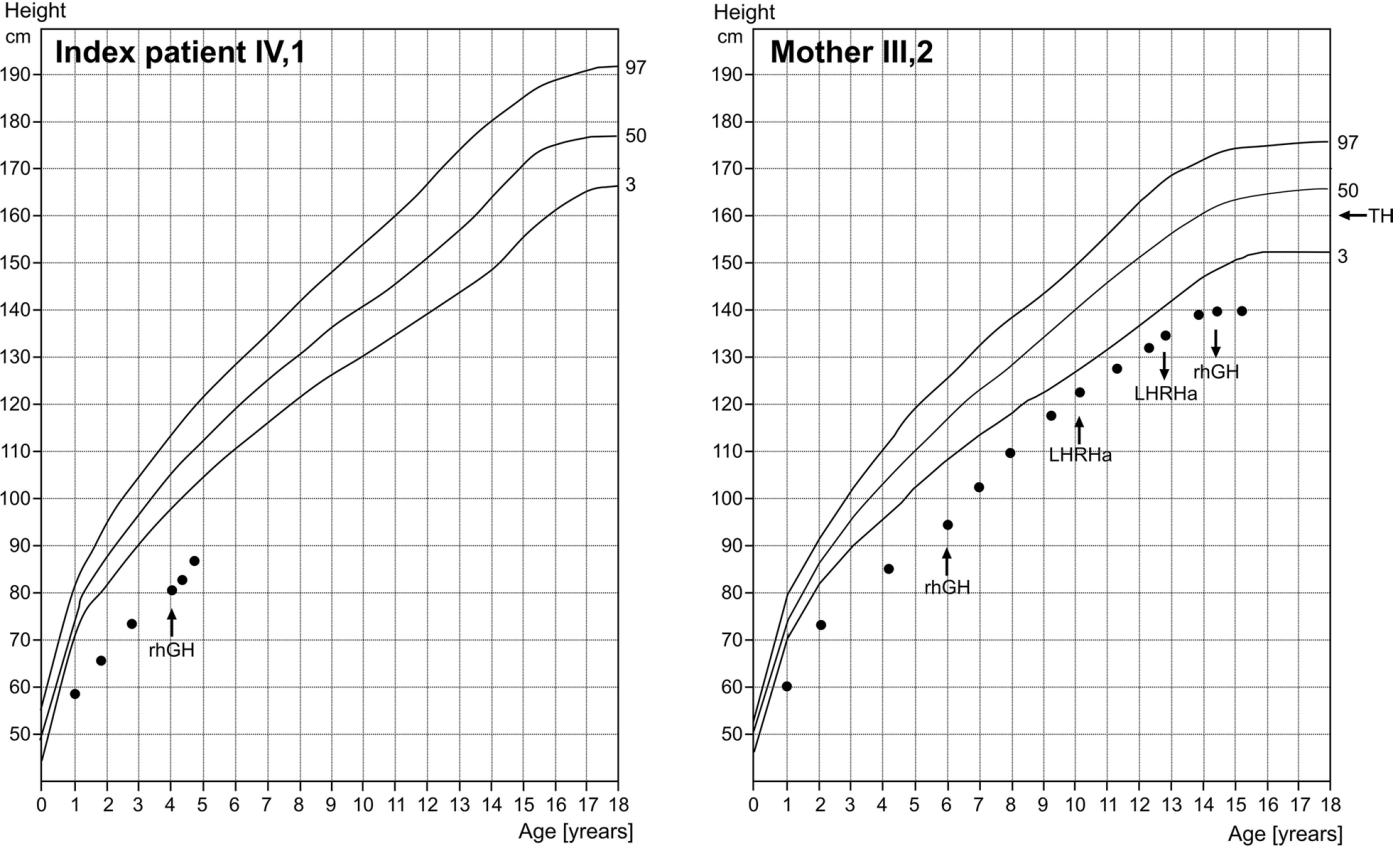
Growth charts of index patient and his affected mother. The percentiles shown are according to Prader et al. [18]. The “rhGH” arrows indicate the start and the cessation of treatment with recombinant GH. The “LHRHa” arrows indicate the start and the cessation of treatment with an LHRH agonist. The “TH” arrow indicates the target height of the mother. The index patient’s target height could not be determined, because his mother is affected

The index patient presented first to us at the age of 22 months. He had relative macrocephaly with a prominent forehead, a triangular face with a very small chin, low-set, protruding and retroverted ears, but no body asymmetry (Fig. 2; at the age of 4.1 years). His length was 65.6 cm (− 5.9 SDS) and his weight 5.3 kg (BMI: − 4.4 SDS). His mental development status was normal. His postnatal height velocity was low, without catch-up growth (Fig. 3). Serum insulin-like growth factor-1 (IGF-1) was low–normal with 58 ng/ml (− 1.63 SDS), and insulin-like growth factor-1 binding protein-3 (IGFBP-3) was 2.332 ng/ml (− 0.98 SDS).

Treatment with recombinant human GH (rhGH) was started at the age of 4.1 years. At that time, blood analysis revealed normal values for cortisol, IGF-1 and IGF-2. Now, after 7.5 months of treatment, growth response was good with a height velocity of 9.9 cm/year (Fig. 3), which corresponds to + 0.25 studentized residuals based on an SGA reference group treated with rhGH [19].

The affected mother (III,2) was diagnosed with SRS at the age of 1.1 years based on intrauterine growth retardation (term birth length 41 cm, − 5.4 SDS; birth weight 1560 g, − 5.2 SDS), relative macrocephaly (head circumference at 18 months of age 44.3 cm, − 2.4 SDS), prominent forehead, early feeding difficulties, failure to thrive and low BMI. Serum IGF-1 was 109 ng/ml (0.02 SDS) and IGFBP-3 was 3577 ng/ml (0.44 SDS), which was quite normal. Stimulated GH secretion was high–normal with a peak of 29.6 ng/ml after arginine challenge. Spontaneous nocturnal GH secretion was very high with a mean GH serum concentration of 13.1 ng/ml and peaks of 50.8, 17.9 and 22.5 ng/ml (blood collection from 8.30 p.m. to 8.00 a.m.). There were no clinical signs of adrenal insufficiency; in contrast, adrenarche with pubarche started precociously at the age of 6.8 years.

Treatment with rhGH was started at the age of 6.0 years, when bone age was 4.2 years. At that time, her height was 94.7 cm (− 5.0 SDS). Response to rhGH (60 µg/kg*d) was moderate with first year growth velocity of 8.8 cm/year, which corresponds to − 0.92 studentized residuals based on an SGA reference group treated with rhGH [19]. Figure 3 depicts the growth chart of the mother of the index patient. Her puberty began at the age of 10.2 years and was blocked by the use of the GnRH agonist Leuprorelin for 2.5 years. Her menarche age was 13.5 years. GH treatment was stopped at the age of 14.3 years when she reached an adult height of 140 cm (− 4.2 SDS) (Fig. 2). The gain in height was 0.8 SDS over the whole treatment period. The maternal grandmother (II,2) has an adult height of 158.8 cm and no signs of SRS (Fig. 2).

## Discussion

This is the third familial case of SRS associated with a missense mutation of the PCNA-binding domain of *CDKN1C*. The pedigree of the reported family and the genetic findings of the members clearly support a causative relationship. Adrenal insufficiency was absent in mutation carriers excluding IMAGe syndrome, whose causative missense mutations cluster within the same region of *CDKN1C*. The phenotype of the index patient and his mother is explained by maternal inheritance of the pathogenic *CDKN1C* variant. The absence of Silver–Russell syndrome in the maternal grandmother could be due to inheritance of the mutant allele from the great-grandfather, but the great-grandfather is not a carrier of the variant. We speculate that the change most likely occurred de novo on the paternal allele. Though functional studies of the novel *CDKN1C* mutation p.(Arg279Ser) were not conducted, the functional data from Brioude et al. of the variant NM_000076.2: c.836G>T, p.Arg279Leu obtained from HEK293 cells suggested increased protein stability as the mechanism conferring gain-of-function [4]. Similarly, the in vitro analysis of a novel *CDKN1C* variant p.(Arg316Gln) detected in a sporadic case with SRS from Japan, which affects an amino acid at the very end of the PCNA domain, suggested increased protein stability as the biochemical mechanism [6].

Body asymmetry, a frequent characteristic in SRS caused by mosaic IC1 hypomethylation, was absent in the index patient and his affected mother. This is in agreement with the previous clinical findings in patients with the p.Arg279Leu variant [4, 5], the sporadic case from Japan [6] and other patients with SRS and genomic mutations of *IGF2* which were not mosaic [20–24]. In agreement with a previous report [5], IGF-1 serum levels were normal in the index patient and his mother. The mother of the index patient had increased nocturnal GH secretion, which may resemble compensatory hypersecretion.

The efficacy of the mother’s treatment with rhGH for eight years and the GnRH agonist leuprorelin for 2.5 years was moderate with a total gain in height SDS of just 0.8. A similar outcome was reported for a French girl with SRS due to the *CDKN1C* missense mutation p.Arg279Leu, who gained approximately 0.7 height SDS during five years of rhGH treatment [4]. A much better outcome had her affected sister, who gained approximately 2.5 height SDS. She was started early on rhGH and was treated for 9 years [4]. Her early puberty was treated with the GnRH agonist triptorelin for 2.5 years and with cyproterone acetate for further 3 years [4]. These individual outcomes reflect the variability of treatment responses we observe in Silver–Russell syndrome [25].

Since the first report of a genomic *IGF2* mutation in familial SRS by Begemann et al. [12], eleven additional *IGF2* mutations in only sporadic cases were reported [13, 20–24]. This number is not high, but higher than the three families and one sporadic case reported with genomic *CDKN1C* mutations and SRS ([4–6], this study). It is unlikely that a technical bias hinders the detection of *CDKN1C* mutations in SRS. A possible explanation of the different prevalence is the qualitative difference between loss-of-function (*IGF2*) and gain-of-function mutations (*CDKN1C*). The spectrum of *CDKN1C* mutations causing SRS seems to be restricted to two amino acids of the protein. In contrast, mutations causing *IGF2* loss-of function and SRS scatter through the whole gene encompassing different types of mutations (nonsense, missense, deletion, insertion, frame shift). This qualitative difference is likely to implicate a higher probability for the occurrence of loss-of-function mutations in *IGF2*.

With respect to *CDKN1C* variants and intrauterine growth retardation, the current literature reports now a total of 12 cases with IMAGe syndrome and adrenal insufficiency, comprehensively summarized by Suntharalingham et al. [26], 4 cases with SRS ([4, 5, 14], and this study) and a single case with an undefined short stature syndrome with early manifestation of diabetes mellitus [6]. Variants causing IMAGe syndrome affected codons 272, 274, 276, 278 and 279 of the PCNA domain, while variants found in SRS changed codons 279 and 316 and were therefore located toward the carboxy-terminal region of the PCNA domain [26]. The same was true for the family with the undefined short stature syndrome and early adult onset diabetes mellitus with a codon 281 variant [6]. This analysis suggests that the genomic location of the variant and the type of missense mutation defines the phenotype [26]. The severity of intrauterine growth restriction in IMAGe syndrome reported in the literature ranged from − 2.5 to − 3.8 birth weight SDS and was not different to SRS caused by CDKN1C mutations with a range from − 2.5 to − 5.2 birth weight SDS. In all instances of *CDKN1C* variants experimentally studied, increased stability of the protein was found in vitro.

Based on the available data, it is recommended to perform molecular analysis of *CDKN1C* together with *IGF2, HGMA2* and *PLAG1* in patients with SRS that were negative for the frequent epigenetic disruptions including IC1 hypomethylation. In all cases with functional *CDKN1C* variants, adrenal insufficiency should be excluded.

## Conclusions

In conclusion, familial SRS is rarely caused by gain-of-function mutations in *CDKN1C,* which seems to cluster to a very narrow region. The phenotype resembles SRS without body asymmetry.

## Materials and methods

Clinical data were extracted from the clinical file records or requested from the family members. Birth parameters are given in SDS according to Niklasson et al. [27]. Auxological data of childhood and adolescence are given in SDS according to Prader et al. [18].

Exome sequencing was performed on genomic DNA from the index patient and his affected mother. In brief, exonic regions were enriched with a SureSelect Human All Exon Kit V6 (Agilent technologies, Santa Clara, California) and sequenced as 2 × 125 bp paired-end reads on an HiSeq2500 system (Illumina, San Diego, California). Generated sequence data were analyzed with the megSAP pipeline (https://github.com/imgag/megSAP). Prioritization of disease-associated variants was conducted according to an in-house standard operating procedure and included different filtering steps including the allele frequency of identified variants (e.g., MAF < 0.1% in 1000 g, ExAC or gnomAD, in-house database) as well as predicted effects on protein level. Variant confirmation and testing of additional family members were done by Sanger sequencing. Primers and PCR conditions are available upon request.

## Data Availability

The datasets used and/or analyzed during the current study are available from the corresponding author on reasonable request.

## Abbreviations

CDKN1C: Cyclin-dependent kinase inhibitor 1C
BWSp: Beckwith–Wiedemann spectrum
SRS: Silver–Russell syndrome
IC: Imprinting centers
upd(7)mat: Uniparental disomy of chromosome 7
SDS: Standard deviation score
IGF-1: Insulin-like growth factor-1
IGF-2: Insulin-like growth factor-2
IGFBP-3: Insulin-like growth factor-1 binding protein-3
rhGH: Recombinant human GH
